# Domain Shift in Breast DCE-MRI Tumor Segmentation: A Balanced LoCoCV Study on the MAMA-MIA Dataset

**DOI:** 10.3390/diagnostics16020362

**Published:** 2026-01-22

**Authors:** Munid Alanazi, Bader Alsharif

**Affiliations:** 1 Business Informatics Department, College of Business, King Khalid University, Abha 61421, Saudi Arabia; malanazi@kku.edu.sa; 2 Department of Electrical Engineering and Computer Science, Florida Atlantic University, 777 Glades Road, Boca Raton, FL 33431, USA

**Keywords:** breast cancer, DCE-MRI, tumor segmentation, MAMA-MIA dataset, multi-center learning, domain shift, leave-one-center-out cross-validation (LoCoCV), deep learning, U-Net

## Abstract

**Background and Objectives:** Accurate breast tumor segmentation in dynamic contrast-enhanced MRI (DCE-MRI) is crucial for treatment planning, therapy monitoring, and quantitative studies of breast cancer response. However, deep learning models often have worse performance when applied to new hospitals because scanner hardware, acquisition protocols, and patient populations differ from those in the training data. This study investigates how such center-related domain shift affects automated breast DCE-MRI tumor segmentation on the multi-center MAMA-MIA dataset. **Methods:** We trained a standard 3D U-Net for primary tumor segmentation under two evaluation settings. First, we constructed a random patient-wise split that mixes cases from the three main MAMA-MIA center groups (ISPY2, DUKE, NACT) and used this as an in-distribution reference. Second, we designed a balanced leave-one-center-out cross-validation (LoCoCV) protocol in which each center is held out in turn, while training, validation, and test sets are matched in size across folds. Performance was assessed using the Dice similarity coefficient, 95th percentile Hausdorff distance (HD95), sensitivity, specificity, and related overlap measures. **Results:** On the mixed-center random split, the best three-channel model achieved a mean Dice of about 0.68 and a mean HD95 of about 19.7 mm on the held-out test set, indicating good volumetric overlap and boundary accuracy when training and test distributions match. Under balanced LoCoCV, the one-channel model reached a mean Dice of about 0.45 and a mean HD95 of about 41 mm on unseen centers, with similar averages for the three-channel variant. Compared with the random split baseline, Dice and sensitivity decreased, while HD95 nearly doubled, showing that boundary errors become larger and segmentations less reliable when the model is applied to new centers. **Conclusions:** A model that performs well on mixed-center random splits can still suffer a substantial loss of accuracy on completely unseen institutions. The balanced LoCoCV design makes this out-of-distribution penalty visible by separating center-related effects from sample size effects. These findings highlight the need for robust multi-center training strategies and explicit cross-center validation before deploying breast DCE-MRI segmentation models in clinical practice.

## 1. Introduction

Breast cancer remains one of the leading causes of cancer-related illness and death in women worldwide. This creates a strong need for imaging tools that support accurate diagnosis, careful treatment planning, and the reliable monitoring of therapy response. DCE-MRI plays a central role in this setting. By capturing how contrast agent washes into and out of tissue over time, DCE-MRI provides information about tumor vascularity and enhancement patterns that reflect underlying tumor biology [1].

In clinical practice, breast DCE-MRI is used in several key decisions, such as evaluating response to neoadjuvant chemotherapy, planning breast-conserving surgery or mastectomy, and designing precision radiation therapy fields. For all of these tasks, accurate tumor segmentation is essential. Reliable 3D masks of the tumor volume enable the quantitative assessment of tumor size and shrinkage over time, extraction of enhancement-based biomarkers, and separation of residual disease from post-treatment changes such as fibrosis or edema [2].

Traditionally, tumor delineation is performed manually by expert radiologists. Although this is still considered the gold standard, it has important limitations. Manual contouring is time-consuming, especially in 3D volumes and multi-phase DCE acquisitions. It is expensive because it depends on highly trained experts, and it is subject to inter- and intra-reader variability, which can affect the reproducibility of downstream analyses [3].

Because of these challenges, there has been strong interest in automating breast tumor segmentation using deep learning. U-Net and related convolutional neural network architectures have become the dominant approach to medical image segmentation. In breast DCE-MRI, 3D U-Net variants can capture spatial context across slices and often report high Dice scores on internal validation sets. When training and testing data come from the same institution and similar protocols, these models can reach a performance level that appears close to expert-level [4].

However, success on internal test sets does not guarantee safe performance in real clinical deployment. A model trained at one hospital may perform worse when applied to another, even if the clinical task is identical. This problem is often referred to as domain shift. In breast DCE-MRI, domain shift arises from many practical and unavoidable sources, such as scanner vendor and field strength, hardware and coil design, protocol parameters (for example, timing, spatial resolution, and sequence type), reconstruction software versions, and patient positioning or motion [5].

Unlike CT, MRI does not have standardized intensity units. As a result, the same tissue can look very different across scanners and hospitals. These differences change the distribution of the images seen by the model. A network that has learned scanner-specific appearance patterns at one site may fail to generalize when those patterns change at another site. For automated segmentation systems, this creates a real risk: performance that appears strong in internal experiments may drop in routine multi-center deployment [6].

Despite this risk, many existing studies mainly use random train–test splits that mix scans from all participating centers. This setup is useful for initial benchmarking, because it maximizes the amount of data available for training and produces stable estimates of in-distribution accuracy. However, it also makes the test set statistically similar to the training set. In this case, the evaluation does not fully answer the following clinically important question: if a model is trained on data from several hospitals, how well will it work on a new hospital that was never seen during training? Answering this question requires explicit out-of-distribution testing. In the context of multi-center imaging, this means keeping entire centers unseen during training and then measuring performance on those centers. Without such an evaluation, it is easy to overestimate how robust a model really is when moved to a new site [7].

The MAMA-MIA breast DCE-MRI dataset is a suitable resource for studying this problem. It includes more than 1500 cases with expert-validated 3D tumor segmentations and rich metadata describing acquisition conditions. Importantly, the dataset is organized into distinct institutional groups that reflect different acquisition environments. In this work, we focus on three major groups: ISPY2, DUKE, and NACT. Each group corresponds to a different combination of hardware, protocols, and patient populations. This diversity makes MAMA-MIA well suited for measuring center-wise generalization and quantifying how strong domain shift can be in breast DCE-MRI tumor segmentation [8].

At the same time, the design of the evaluation protocol also matters. Simple leave-one-center-out experiments can be affected by imbalanced sample sizes across centers. For example, if one center has far fewer cases, a naive split can make a model look worse on that center simply because there was less training data, not because the domain itself is harder. To avoid this confounding effect, it is important to control for sample size when comparing performance across centers.

In this work, we perform a domain shift analysis of breast DCE-MRI tumor segmentation using a balanced leave-one-center-out cross-validation (LoCoCV) design on the MAMA-MIA dataset. We deliberately use a standard 3D U-Net as a strong, well-established baseline architecture, rather than proposing a new model. This choice keeps this study focused on how evaluation design and input representation affect performance under domain shift, instead of mixing these effects with architectural novelty. First, we establish an in-distribution baseline using a random patient-wise split that mixes cases from all centers. This represents the typical evaluation used in many prior works. Second, we perform LoCoCV across the three institutional groups (ISPY2, DUKE, NACT). In each fold, two centers are used for training and validation, and the remaining center is held out entirely for testing. Third, we design the LoCoCV folds to be balanced, with equal numbers of training, validation, and test cases across folds, so that differences in performance are not driven by sample size imbalance.

We report performance using the Dice similarity coefficient (DSC), 95th percentile Hausdorff distance (HD95), sensitivity, and specificity. These metrics provide complementary information: Dice reflects volumetric overlap, HD95 captures boundary errors, and sensitivity and specificity reflect the balance between detecting tumor and avoiding false positives. By comparing the random split baseline with each LoCoCV fold, we directly measure the out-of-distribution penalty caused by center shift and examine how it varies across institutions.

The main contributions of this work are as follows:

- A systematic analysis of domain shift in breast DCE-MRI tumor segmentation using a large multi-center dataset with expert tumor masks.
- A balanced leave-one-center-out cross-validation protocol on the MAMA-MIA dataset that separates domain effects from sample size effects.
- A clear comparison between in-distribution performance (random patient-wise splits) and out-of-distribution performance (unseen centers), using clinically interpretable metrics.
- A deliberately simple 3D U-Net baseline that provides a transparent reference for future work on more advanced architectures, domain adaptation methods, and harmonization strategies.

Overall, this study aims to provide transparent evidence of how domain shift affects breast DCE-MRI tumor segmentation and to highlight the importance of reliable multi-center training and validation before deploying such models in real clinical environments.

## 2. Related Work

### 2.1. Deep Learning for Breast DCE-MRI Segmentation

Early work on breast MRI often focused on semi-automatic or rule-based methods that relied on intensity thresholds, region growing, or deformable models for breast and lesion segmentation. These approaches improved reproducibility compared with purely manual contouring, but they still required substantial user interaction and were sensitive to noise and acquisition parameters. For example, Ertas et al. proposed a computerized volumetric segmentation pipeline for breast tissue and lesion analysis across multiple centers, but this method combined several hand-crafted steps and showed limited robustness across different scanners and protocols [9].

With the rise in deep learning, U-Net-type architectures have become the dominant approach to medical image segmentation, including breast DCE-MRI. Several studies have shown that convolutional neural networks can reach near-expert performance on internal datasets when training and test data come from the same distribution. Khaled et al. developed a U-Net ensemble for lesion segmentation in breast DCE-MRI, combining multiple models with different input representations of the dynamic series. Their ensemble consistently outperformed individual models and earlier methods on a single center dataset, illustrating the benefit of learning complementary features from the time course of contrast enhancement [10].

Douglas et al. investigated U-Net-based mass segmentation on early post-contrast subtraction images and reported promising Dice scores for both mass and non-mass enhancing lesions when training and testing were performed on data from the same institution [11]. Park et al. studied 3D breast cancer segmentation in DCE-MRI with weak annotation, showing that deep models can still achieve good tumor segmentation performance when only sparse labels or coarse masks are available. This is important given the cost of full voxel-wise annotation in large volumetric DCE-MRI datasets [12].

Beyond pure tumor masks, some work has used segmentation as part of larger analysis pipelines. Jiao et al. presented a deep convolutional framework that jointly performs breast segmentation and mass detection in DCE-MRI, highlighting how reliable lesion masks can support computer-aided diagnosis and quantitative biomarker extraction [13]. Other studies have targeted whole breast segmentation to support volumetric density assessment and quantitative breast analysis. Narimani et al. compared several deep learning architectures for automatic breast region segmentation and reported that deep models can reduce manual effort while maintaining high overlap metrics, although their evaluation focused mainly on internal generalization [14].

Nowakowska et al. proposed an attention U-Net for the segmentation of fibroglandular tissue and background parenchymal enhancement in breast DCE-MRI, demonstrating that attention mechanisms can improve the segmentation of parenchymal structures that are important for background parenchymal enhancement analysis [15]. Their work also included some tests on external data, suggesting a degree of robustness to scanner changes, but the primary focus was parenchymal tissue rather than primary tumor segmentation.

Taken together, these studies show that deep learning, in particular U-Net-based architectures, can deliver high segmentation accuracy for breast DCE-MRI when evaluated on internal splits or on data that are closely matched to the training distribution. However, most of this work has been conducted on single-center datasets or on multi-center cohorts that are treated as a single pooled domain. A systematic analysis of how these models behave when applied to unseen centers, under controlled and balanced center-wise evaluation, is still limited.

### 2.2. Multi-Center Breast MRI and Domain Shift

Domain shift is increasingly recognized as a key barrier to the safe deployment of artificial intelligence in medical imaging. For breast MRI, differences in scanner vendor, field strength, coil configuration, protocol parameters, reconstruction pipelines, and patient population can all change the distribution of voxel intensities and contrast dynamics. A model that performs well on data from one site may fail when deployed at another, even if the clinical task is the same.

Several recent works have started to address domain shift in breast MRI segmentation. Kuang et al. introduced MSCDA, a multi-level semantic-guided contrastive domain adaptation framework for cross-domain breast MRI segmentation [16]. Their method combines self-training and contrastive learning to align feature distributions between a labeled source domain and an unlabeled target domain and was validated in cross-domain settings where the source and target cohorts differ in population or acquisition conditions. The results showed that domain adaptation can substantially improve performance on the target domain compared with naive direct transfer.

Nowakowska et al. evaluated their attention U-Net model for fibroglandular tissue and background parenchymal enhancement on data from a different field strength and an external institution and reported that the model maintained acceptable performance despite changes in scanner and protocol [15]. Osuala et al. explored a complementary direction by using conditional generative models to simulate dynamic tumor enhancement patterns in breast MRI, showing that synthetic DCE-MRI data can be used to augment training sets and improve robustness to variations in contrast dynamics [17].

These studies share the goal of improving cross-domain performance using advanced architectures or domain adaptation strategies. However, they typically involve a limited number of source and target domains and often focus on specific segmentation tasks such as parenchymal tissue or whole breast segmentation rather than detailed primary tumor segmentation. In addition, many works still rely on random splits within each domain and do not fully separate the effect of domain shift from the effect of training sample size or class balance.

### 2.3. Multi-Center Benchmarks and the MAMA-MIA Dataset

A major obstacle to studying domain shift in breast DCE-MRI segmentation has been the lack of large multi-center datasets with expert tumor masks. Most earlier datasets contained tens of to a few hundred cases from one or two institutions, which is enough to demonstrate internal performance but not enough to systematically analyze center-wise generalization. Other breast MRI datasets exist in the literature, but they usually provide only single-center data, lack full DCE series with 3D tumor masks, or include too few centers to support a balanced leave-one-center-out design, so they were not suitable for the present analysis.

The MAMA-MIA dataset was created to address this gap. Garrucho et al. introduced MAMA-MIA as a large-scale multi-center breast cancer DCE-MRI benchmark that includes 1506 pre-treatment cases collected from multiple public collections in The Cancer Imaging Archive, with expert segmentations of primary tumors and non-mass enhancement areas for more than 400 cases [8]. The dataset is organized into institutional groups that reflect different acquisition environments and scanner configurations and is accompanied by detailed metadata and standardized preprocessing for research use.

The MAMA-MIA team has also organized an open challenge to benchmark segmentation and downstream prediction methods on this dataset, providing baseline models such as nnU-Net to facilitate reproducible research and comparison between approaches [8]. The challenge design emphasizes real-world clinical variability by including multi-vendor and multi-center DCE-MRI with varying temporal resolution and contrast timing.

Most early uses of MAMA-MIA have focused on reporting overall performance across the full dataset or on demonstrating new architectures that can attain high Dice scores when trained and evaluated with standard random splits. This is an important first step, but it does not fully answer how robust these models are when tested on centers that were not seen during training. The dataset design naturally supports center-wise analysis, but a systematic and balanced leave-one-center-out evaluation of primary tumor segmentation across the major institutional groups has not yet been fully explored. A detailed description of how MAMA-MIA is used in this work, including inclusion criteria and preprocessing, is provided in the Section 3.

### 2.4. Cross-Validation Strategies for Multi-Center Evaluation

How models are evaluated is as important as the choice of architecture or dataset. Cross-validation strategies in artificial intelligence for medical imaging were reviewed in detail by Bradshaw et al., who emphasized that the cross-validation scheme must reflect the intended deployment scenario [18]. Random k-fold splits at the image or slice level are appropriate for estimating in-distribution performance, but they can be misleading when the goal is deployment across sites, scanners, or time periods.

For multi-center data, more stringent strategies such as leave-one-center-out or leave-one-source-out cross-validation have been proposed. Pauli et al. studied balanced leave-one-subject-out cross-validation and showed that balancing the folds is important to avoid confounding performance with differences in sample size and class distribution [19]. In segmentation, frameworks such as MIScnn support k-fold and leave-one-out schemes, but many medical image segmentation studies still adopt random patient-wise splits that mix all centers together [20]. This design maximizes data usage and is convenient for model development, but it does not isolate the effect of domain shift. The metrics obtained from mixed-center cross-validation may therefore overestimate robustness when a model is deployed at new institutions.

In summary (Table 1), prior research has established that deep learning can achieve strong segmentation performance in breast DCE-MRI and that domain adaptation and multi-center datasets can help address domain shift. Most single-center DCE-MRI tumor segmentation studies report internal Dice scores in the range of approximately 0.70–0.80 for primary lesions when training and testing are performed on data from the same institution [10,11,12]. In our study, the random split baseline on the multi-center MAMA-MIA dataset attains comparable center-wise Dice values (e.g., 0.73 at ISPY2 and around 0.60 at DUKE and NACT for the three-channel model), indicating that a standard 3D U-Net can match the level of accuracy of recent methods when evaluated under similar mixed-center conditions. However, when we move to a balanced leave-one-center-out design, Dice drops to roughly 0.40–0.48 on held-out centers, and HD95 increases, revealing a much larger out-of-distribution penalty than is typically visible in prior work. The present study therefore complements existing methods by quantifying this center-wise performance drop on MAMA-MIA and by isolating the effect of domain shift itself, rather than proposing yet another architecture or adaptation strategy.

Beyond voxel-wise breast MRI segmentation, there is a broader trend toward foundation-style models and training schemes in medical image analysis. Recent surveys on vision-language models in medical imaging [21] and on knowledge distillation and teacher–student learning [22] summarize how large multi-modal models, prompting, and distillation can transfer knowledge across tasks, modalities, and institutions. These approaches are highly relevant to future work on robust breast DCE-MRI segmentation. In this study we deliberately focus on a conventional supervised 3D U-Net trained on MAMA-MIA, providing a transparent, center-wise baseline. The balanced LoCoCV analysis presented here can serve as a reference protocol against which future vision-language or distillation-based models can be evaluated when the goal is reliable multi-center deployment.

## 3. Materials and Methods

### 3.1. Dataset and Random Split

We used the public multi-center MAMA-MIA breast DCE-MRI dataset, which contains lesions from three main centers: DUKE, ISPY2, and NACT. After quality control, we retained a total of 1506 lesions. For each lesion, three temporal phases were available (pre-contrast, early post-contrast, and late post-contrast). The dataset provides 3D voxel-wise annotations of the primary enhancing tumor together with center labels and acquisition metadata. This makes it suitable for studying multi-center domain shift in breast DCE-MRI tumor segmentation.

For the segmentation experiments reported in this paper, we drew a stratified subset of 1454 lesions from the 1506 available cases. This subset was used for both the random split baseline and the balanced leave-one-center-out (LoCoCV) experiments. We then constructed a random, center-stratified split at the lesion level with 1054 lesions for training, 200 for validation, and 200 for testing. This corresponds to roughly 70%/15%/15% of the selected lesions. The remaining 52 lesions were not included in these experiments and were reserved for auxiliary analyses.

The random split was stratified so that all three centers (DUKE, ISPY2, NACT) were represented in each subset. This split represents a best-case in-distribution scenario where training and testing data come from the same set of centers and therefore provides an upper bound on performance before we study cross-center domain shift.

For every lesion, we stored the following fields in a metadata table (mama_mia_meta.csv):

- case_id: Unique case identifier.
- img_path: Path to the selected 3D DCE-MRI volume.
- mask_path: Path to the corresponding 3D tumor mask.
- center_id: Site label (DUKE, ISPY2, or NACT).

The raw MAMA-MIA data are provided as NIfTI volumes stored in separate folders for images and segmentations, with multiple DCE phases per case. We implemented a Python 3.9.16 utility that scans the image and segmentation directories, matches images and masks by a shared case identifier, infers the center_id from the folder structure or filename prefix, and writes the metadata table with one representative 3D image and one tumor mask per case.

### 3.2. Input Representations: 1-Channel vs. 3-Channel

We compared two input configurations for the 3D segmentation network.

One-channel (1ch) model.

- Input: A single post-contrast volume (first post-contrast time point).
- Shape after preprocessing: [1,1,128,128,128] (batch, channels, *z*, *y*, *x*).

Three-channel (3ch) model.

- Input: Three temporal phases stacked along the channel dimension:
  – Channel 1: Pre-contrast.
  – Channel 2: Early post-contrast.
  – Channel 3: Late post-contrast.
- Shape after preprocessing: [1,3,128,128,128].

The network architecture, loss function, optimizer, and training schedule are identical for the 1ch and 3ch models. Only the number of input channels differs.

### 3.3. Preprocessing

All preprocessing was implemented using MONAI version: 1.5.1 dictionary transforms. For each case, we applied the following steps:

- Load the 3D image volume and tumor mask.
- Convert both the image and mask to channel-first format.
- Reorient to RAS axis convention.
- Resample the image and mask to a common voxel spacing of (2.0,0.8,0.8)mm.
- Per-volume intensity normalization: Scale intensities between the 1st and 99th percentiles into the range [0,1] to reduce scanner-specific intensity differences.
- Resize with symmetric padding or cropping to a fixed region of interest of 128×128×128 voxels.
- Convert to MONAI meta-tensors.

During both training and inference, the network processes 3D patches of size 128×128×128 voxels. At inference time, we use sliding-window inference with an overlap of 0.5 in each spatial direction and aggregate patch predictions back into a full-volume segmentation. This strategy allows us to train 3D models on harmonized high-resolution volumes within GPU memory limits.

### 3.4. Network Architecture

We used a 3D U-Net implemented in MONAI (monai.networks.nets.UNet) as the backbone. The main settings are as follows:

- Input channels: 1 (1ch model) or 3 (3ch model).
- Output channels: 2 (background and tumor).
- Encoder and decoder: Convolutional blocks with instance normalization and non-linear activations.
- Fully convolutional operation on 128×128×128 input patches.

The network follows a standard encoder–decoder structure with skip connections between matching resolutions. The encoder path progressively increases feature channels while downsampling spatially, and the decoder path upsamples and fuses encoder features through these skip connections. The final layer is a 1×1×1 convolution that maps decoder features to two output channels, followed by softmax during inference. The architecture is kept fixed across all experiments so that performance differences can be attributed to the input representation and data split rather than model capacity.

We chose this conventional 3D U-Net on purpose, because it is well understood and widely used in medical image segmentation. Using a single fixed backbone across all experiments provides a clean reference for future work and keeps the focus of this study on the evaluation protocol and domain shift.

### 3.5. Training Procedure

Training was implemented in PyTorch (v2.7.1+cu118) CUDA 11.8 and cuDNN 9.1.0. with MONAI. The setup is as follows:

- Optimizer: AdamW with weight decay $10^{-4}$.
- Initial learning rate: $3\times 10^{-4}$.
- Learning rate schedule:
  – Linear warmup over the first 10 epochs.
  – Cosine decay down to $1\times 10^{-6}$ afterwards.
- Batch size: 1 (one 3D patch).
- Maximum epochs: 200.
- Automatic mixed precision (AMP) with gradient scaling.
- Gradient norm clipping at 1.0.

We used a combined Dice–focal loss:

(1) $$L=\alpha \;L_{\mathrm{Dice}}+\beta \;L_{\mathrm{Focal}},$$

with α=0.7, β=0.3, and focal parameter γ=2.0. The loss is computed on softmax outputs with one-hot-encoded labels. The Dice term ignores the background class and focuses on tumor voxels.

Model selection is based on the validation set Dice score. After each epoch, we evaluate the network on the validation subset and save the checkpoint with the best mean validation Dice. This best checkpoint is then used for final evaluation on the held-out test set.

### 3.6. Experimental Design

We designed two complementary experiments to separate in-distribution performance from cross-center generalization.

**Figure 1:**
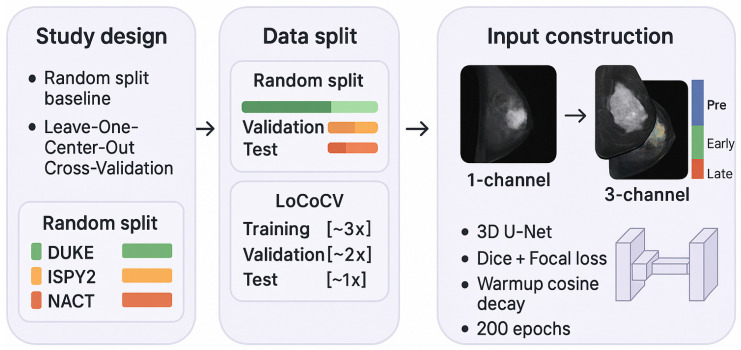
The methodology overview. This study compares a random split baseline and balanced leave-one-center-out cross-validation (LoCoCV). The data split panel shows how training, validation, and test sets are formed in each setting. The input construction panel illustrates the 1-channel model (single post-contrast volume) and the 3-channel model (pre, early, and late DCE phases) that are fed into a shared 3D U-Net with Dice + focal loss, a warmup cosine learning rate schedule, and 200 training epochs.

Figure 1 summarizes the overall study design, data splits, and input constructions used in this work.

#### 3.6.1. Random Split Baseline

The first experiment establishes an in-distribution baseline. Starting from the metadata table, we created a random lesion-wise split that mixes cases from all centers, with each case appearing in exactly one subset. This setting matches typical practice in many prior segmentation studies and provides an optimistic upper bound on performance when training and test data come from the same centers and distribution. The random split is used to conduct the following:

- Train baseline 1ch and 3ch 3D U-Net models with access to data from all centers.
- Tune general training hyperparameters and verify correct implementation.
- Measure per-case Dice and HD95 on a held-out mixed-center test set.

#### 3.6.2. Balanced Leave-One-Center-Out Cross-Validation

To study domain shift across centers, we also consider a balanced leave-one-center-out cross-validation (LoCoCV) design across the three major center groups (DUKE, ISPY2, NACT). For each center group *c*, we define one LoCoCV fold as follows:

- Test set: Only cases from center group *c*.
- Training and validation sets: Cases from the remaining center groups.

The case indices for each LoCoCV fold are generated once using a fixed random seed and stored in the metadata split files that we release with this work. Each lesion appears in the test set of exactly one fold and never in the corresponding training or validation sets, so test samples are independent across folds. We do not repeatedly resample the folds; instead, the balanced design provides a single, deterministic set of splits that can be reproduced exactly and interpreted as three deployment scenarios (one per held-out center).

To avoid confounding center effects with sample size, we construct balanced folds with fixed numbers of training, validation, and test cases. In each fold we sample an equal number of test cases from the held-out center (limited by the smallest center) and fixed numbers of training and validation cases from the remaining centers. This yields three LoCoCV folds in which test cases always come from an unseen center group, while training and validation cases come from the other groups with matched sample sizes. The balanced design allows us to attribute performance differences between folds primarily to domain shift.

### 3.7. Evaluation Protocol and Metrics

For all test evaluations, we run full-volume inference using sliding windows with patch size 128×128×128 voxels and 50% overlap in each spatial direction. Network logits are converted to class probabilities using softmax and then to discrete labels by taking the argmax across channels.

For each test case, we compute the following:

- The Dice similarity coefficient (Dice) for tumor voxels, measuring volumetric overlap between prediction and ground truth while ignoring background.
- The 95th percentile Hausdorff distance (HD95) between predicted and reference tumor surfaces (in millimeters), measuring boundary distance while reducing sensitivity to outliers.
- Sensitivity (recall) on tumor voxels, the fraction of tumor voxels correctly labeled as tumor.
- Specificity on background voxels, the fraction of non-tumor voxels correctly labeled as background.
- The empty prediction rate, the percentage of cases for which the model predicts no tumor.

We then aggregate these metrics as follows:

- Over all cases to obtain overall means, standard deviations, and score distributions.
- Per center (DUKE, ISPY2, NACT) to study center-wise performance and domain shift.

For the random split baseline, we report the mean and standard deviation across all test cases. For the LoCoCV analysis, we compute metrics per case within each fold and then aggregate them by center group, yielding the center-wise mean and standard deviation for Dice, HD95, sensitivity, specificity, and the empty prediction rate.

For each center and metric, we therefore report the mean ± standard deviation together with the number of test lesions, which allows readers to approximate 95% confidence intervals (for example, mean ± 1.96·SD/√n) if desired.

### 3.8. Implementation Details

All experiments share a common codebase that handles metadata creation, configuration, training, and evaluation. A metadata script builds the mama_mia_meta.csv file and the split files for the random split and LoCoCV experiments. Configuration modules define common hyperparameters such as target spacing, patch size, batch size, number of epochs, and AMP usage. Training and evaluation scripts wrap the 3D U-Net model and MONAI data pipelines for both experimental settings. All models are trained and evaluated on GPUs with sufficient memory to process 128×128×128 patches with a batch size of 1.

## 4. Results

### 4.1. Balanced Leave-One-Center-Out (LoCoCV): One-Channel vs. Three-Channel

#### 4.1.1. LoCoCV Center-Wise Quantitative Performance

**Figure 2:**
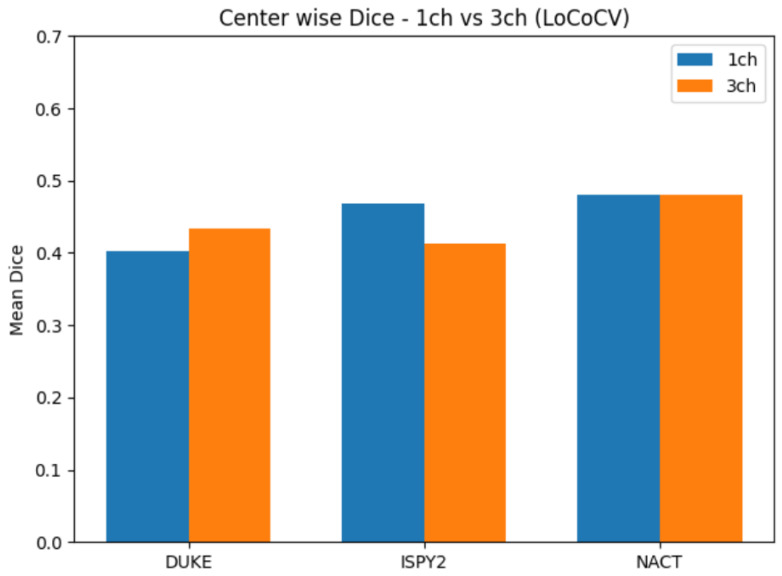
The center-wise mean Dice for the LoCoCV experiment. Bars compare the 1-channel and 3-channel models when each center (DUKE, ISPY2, NACT) is held out for testing.

**Figure 3:**
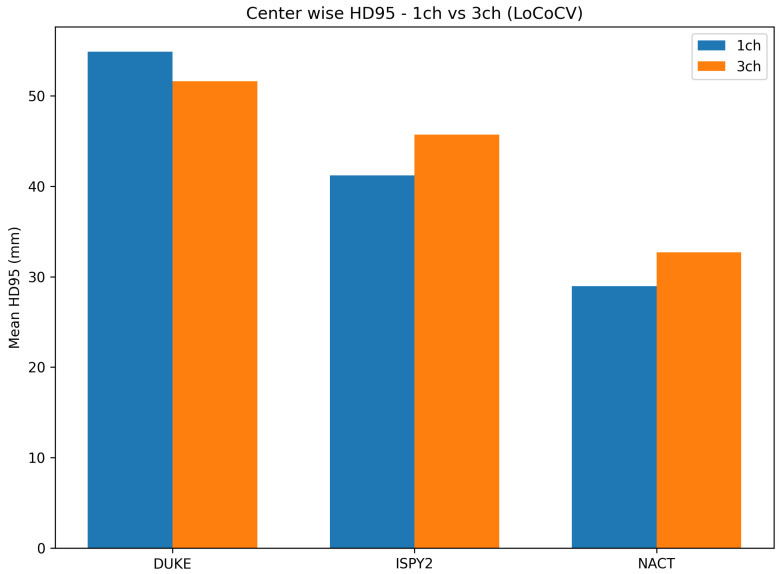
The center-wise mean HD95 (mm) for the LoCoCV experiment. Lower values indicate closer agreement between predicted and reference tumor surfaces on the held-out center.

Table 2 and Figure 2 summarize the center-wise Dice, while Figure 3 summarizes HD95 for the LoCoCV experiment.

The key observations from Table 2 and Figure 2 and Figure 3 are as follows:

- Overall across centers (ALL). When we aggregate all three folds (192 test lesions), the one-channel model reaches a mean Dice of 0.451±0.326 and a mean HD95 of 41.3±38.6 mm. The three-channel model has a very similar mean Dice of 0.442±0.326 and a slightly higher mean HD95 of 43.1±36.4 mm. This shows that, on average, adding three-phase input does not give a clear advantage under center-wise domain shift.
- DUKE held out. The mean Dice improves from 0.402 (1ch) to 0.433 (3ch), and the mean HD95 decreases from 54.9 mm to 51.6 mm. For DUKE, the three-channel model slightly improves in both overlap and boundary accuracy.
- ISPY2 held out. The mean Dice drops from 0.469 (1ch) to 0.413 (3ch), and the mean HD95 increases from 41.2 mm to 45.7 mm. Here the one-channel model is clearly more robust than the three-channel model.
- NACT held out. The mean Dice is almost identical (0.481 vs. 0.480), but the mean HD95 increases from 28.9 mm to 32.7 mm for the three-channel model, which suggests slightly less precise boundaries.

Overall, LoCoCV reveals a marked performance drop compared with the random split baseline (Section 4.2), and the effect of three-channel input is center-dependent: it helps at DUKE, is neutral at NACT, and harms performance at ISPY2. When all centers are pooled, the simpler one-channel representation is at least as competitive as the three-channel variant.

#### 4.1.2. Dice Distributions Across All LoCoCV Folds

**Figure 4:**
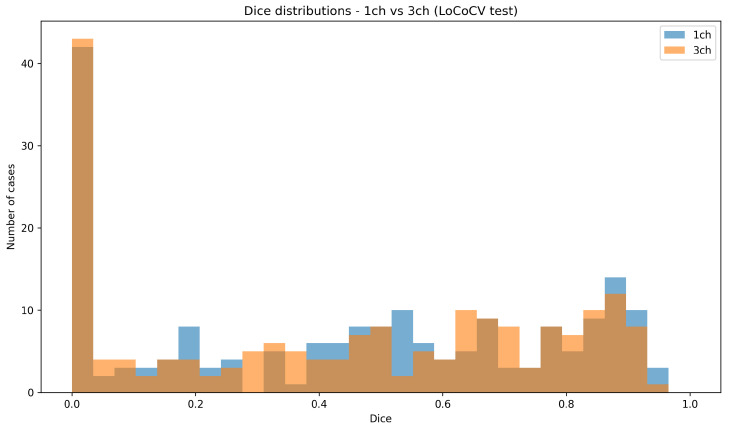
Dice distributions over all LoCoCV test cases for the 1-channel and 3-channel models. Both models show a long left tail of difficult cases, and the overall distributions are similar, with small center-dependent shifts.

Figure 4 compares the Dice distributions for all 192 LoCoCV test cases across the three folds.

- Both models have a long left tail with near-zero Dice for a subset of difficult lesions, which corresponds to near-complete misses in some held-out centers.
- Compared with the random split histogram, the high-Dice region is narrower, and there are more low-Dice cases, confirming that performance is lower and more variable under domain shift.
- The three-channel histogram shows a slight shift toward higher Dice for some cases (e.g., DUKE) but also more failures at others (e.g., ISPY2), consistent with the center-wise averages in Table 2.

These LoCoCV results quantify the out-of-distribution penalty when the model is applied to a new center and provide the main evidence of domain shift in this study.

### 4.2. Random Split Baseline: One-Channel vs. Three-Channel

#### 4.2.1. Center-Wise Quantitative Performance

**Figure 5:**
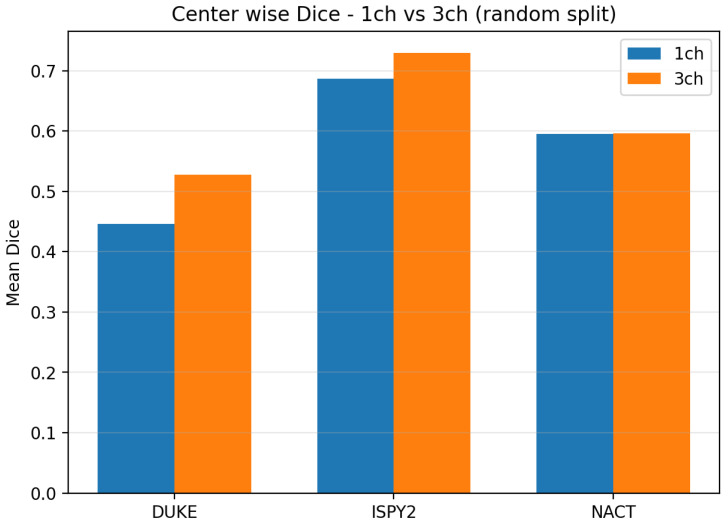
The center-wise mean Dice for the random split experiment. Bars compare the 1-channel and 3-channel models at each center (DUKE, ISPY2, NACT).

**Figure 6:**
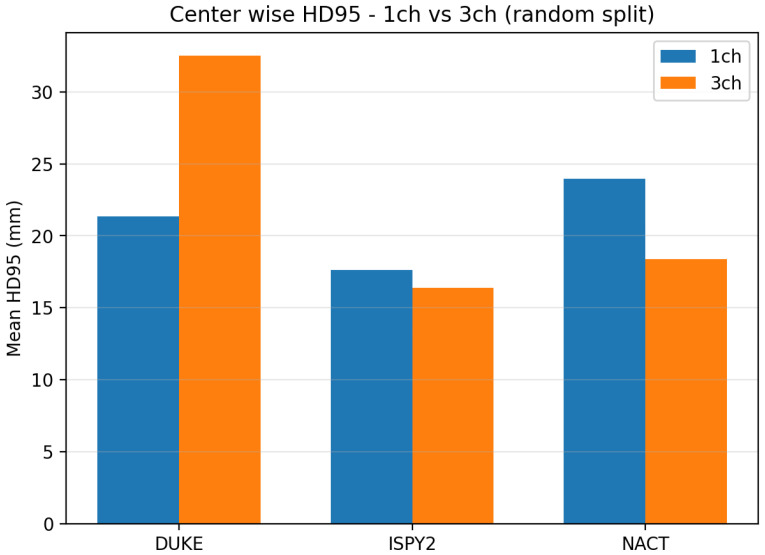
The center-wise mean HD95 (mm) for the random split experiment. Lower values indicate closer agreement between predicted and reference tumor surfaces.

Table 3 and Figure 5 summarize center-wise Dice, and Figure 6 summarizes HD95 for the random split experiment.

The key observations from Table 3 and Figure 5 are as follows:

- DUKE. The mean Dice increases from 0.446 for the one-channel model to 0.528 for the three-channel model, so overlap improves when temporal information is added. At the same time, the mean HD95 increases from 21.3 mm to 32.5 mm, which indicates that a few cases have large boundary errors.
- ISPY2. The mean Dice improves from 0.687 to 0.729, and the mean HD95 decreases slightly from 17.6 mm to 16.4 mm. This means that the three-channel model provides both better volumetric overlap and slightly better boundary accuracy at the largest center.
- NACT. The mean Dice is almost unchanged (0.595 vs. 0.596), but the mean HD95 decreases from 24.0 mm to 18.4 mm. This suggests better boundary placement for NACT, although the test set is small (n=6), so these estimates are less stable.

Overall, on the random split, the following observations can be made:

- The three-channel input improves the mean Dice at the two large centers (DUKE and ISPY2).
- The three-channel input reduces the mean HD95 at ISPY2 and NACT.
- At DUKE, the three-channel model has a higher mean Dice but worse mean HD95, which indicates that a small number of outlier cases strongly affect the distance metric.

Table 4 shows that the three-channel model generally has improved or the same center-wise overlap and detection metrics compared with the one-channel model. For example, at ISPY2, the three-channel Dice/Jaccard increase from 0.720/0.615 to 0.759/0.644, with recall and precision also improving. At DUKE and NACT, the gains are smaller but remain consistent across metrics. These center-wise results support the overall finding that multi-phase input provides a modest but robust benefit under mixed-center conditions.

#### 4.2.2. Dice Distributions

**Figure 7:**
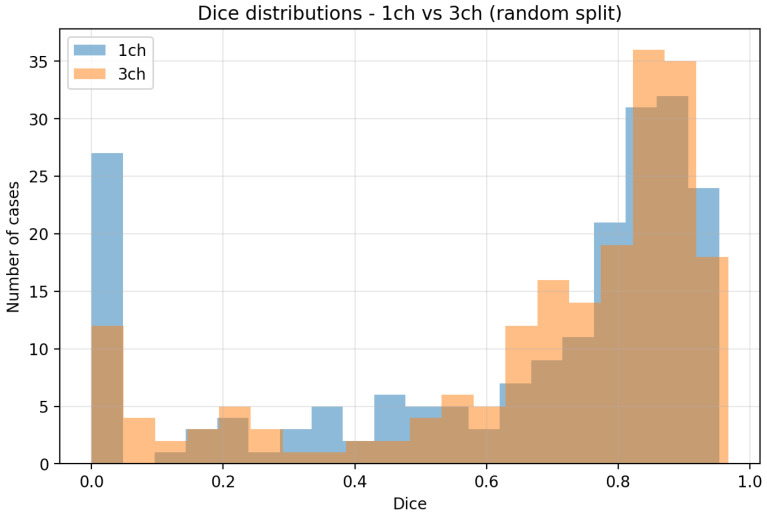
Dice distributions over all random split test cases for the 1-channel and 3-channel models. The 3-channel histogram is shifted toward higher Dice and shows fewer near-zero failures.

Figure 7 compares the Dice distributions for all 200 test cases.

- The one-channel histogram has a long left tail with several cases near zero Dice, which correspond to severe segmentation failures.
- The three-channel histogram is shifted to the right, with more cases above Dice 0.8 and fewer cases with very low Dice.
- The overlap region between the histograms shows that many cases are well segmented by both models, but the three-channel model reduces the number of outliers and increases the proportion of high-quality segmentations.

#### 4.2.3. Additional Overlap and Detection Metrics

Table 5 summarizes additional overlap and detection metrics on the mixed-center random split test set. The three-channel model achieves slightly higher Dice (0.695 vs. 0.662) and Jaccard (0.581 vs. 0.552) than the one-channel model, along with modest gains in recall (0.805 vs. 0.792) and precision (0.681 vs. 0.657). These results confirm that temporal information from multiple DCE phases not only improves volumetric overlap but also increases the fraction of tumor voxels correctly detected, without sacrificing precision.

### 4.3. Qualitative Visual Results

**Figure 8:**
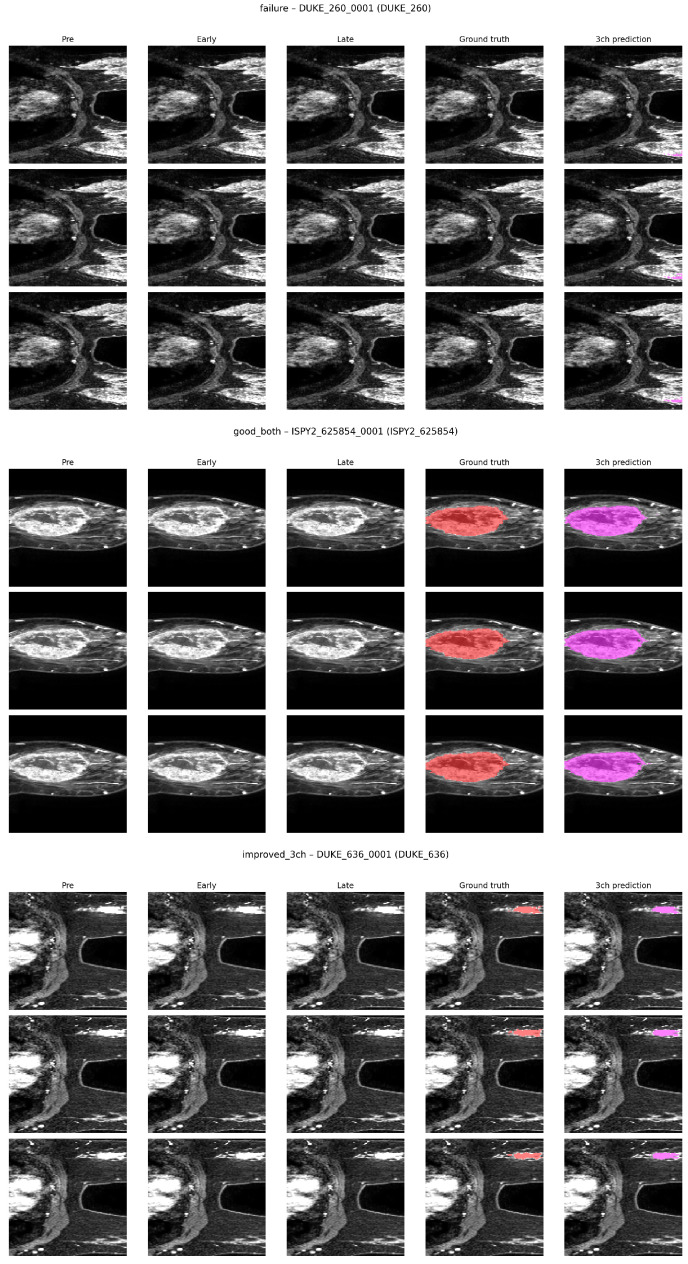
Visual segmentation examples from LoCoCV folds. Rows show axial slices; columns show pre-contrast, early post-contrast, late post-contrast, ground truth (red overlay), and 3ch prediction (purple overlay). (**Top**): Failure case from DUKE (low Dice). (**Middle**): Good case from ISPY2 (high overlap). (**Bottom**): Improved case from DUKE (better boundaries with 3ch input).

To illustrate the model’s performance, Figure 8 shows representative segmentation results for the three-channel model on cases from the DUKE and ISPY2 centers. These include a failure case (low Dice due to under-segmentation), a good case (high overlap with ground truth), and an improved case where the model benefits from multi-phase input. The ground truth is overlaid in red and predictions in purple. Note that performance varies by center, with DUKE showing more boundary errors in failure cases compared to ISPY2.

## 5. Discussion

### 5.1. Impact of Domain Shift on Breast DCE-MRI Segmentation

The main goal of this study was to quantify how much performance is lost when a breast DCE-MRI tumor segmentation model is applied to a center that was not seen during training. The results show a clear gap between in-distribution performance on the mixed-center random split and out-of-distribution performance in the LoCoCV setting.

On the random split, where training and test data come from the same three centers, the three-channel model reaches mean Dice values of 0.528 (DUKE) and 0.729 (ISPY2), with HD95 values close to or below 30 mm at all centers. When the same centers are used as held-out test sets in LoCoCV, the mean Dice drops to the range 0.40–0.48, and the mean HD95 increases to about 41–55 mm (Table 2). The “ALL” LoCoCV row summarizes this shift: a mean Dice of 0.451 (1ch) and 0.442 (3ch) with a mean HD95 around 41–43 mm, clearly worse than the random split baseline.

In practical terms, this means that a model that appears reliable when evaluated on random splits can still underperform when deployed at a new hospital with different scanners and protocols. The drop in Dice and increase in HD95 under LoCoCV indicate that both volumetric overlap and boundary accuracy are strongly affected by center-specific characteristics. In the extended metric set (not shown in detail here), sensitivity tends to decrease more than specificity, which suggests that the dominant failure mode is the under-segmentation of tumor tissue rather than large false positive regions.

An additional finding is that the choice of one-channel versus three-channel input has a smaller effect than the change from random split to LoCoCV. Across all LoCoCV folds, the one-channel and three-channel models have a very similar mean Dice (0.451 vs. 0.442), so domain shift is the main driver of performance loss.

From a statistical perspective, the center-wise Dice differences we observe between the random split and LoCoCV are substantial: drops of 0.10–0.30 are comparable to or larger than the spread of the data within each center, as reflected by the reported standard deviations. This suggests that the effect of domain shift is unlikely to be due to random variation alone. From a clinical perspective, such Dice reductions correspond to noticeable boundary errors and changes in measured tumor volume, especially for small or irregular lesions, which can affect response assessment and eligibility for breast-conserving therapy. In contrast, the differences between the one-channel and three-channel models under LoCoCV are relatively small, and their confidence intervals overlap, so we do not claim a clinically meaningful advantage of one representation over the other in that setting.

### 5.2. Role of Balanced LoCoCV in Revealing Deployment Risk

A key design choice in this work was the use of balanced LoCoCV across the three main center groups. By enforcing equal numbers of training, validation, and test cases in each fold, the evaluation is less likely to confound center effects with differences in sample size.

If one center had far fewer cases than the others and naive splits were used, it would be difficult to tell whether poorer performance on that center was due to stronger domain shift or simply to less training data. The balanced LoCoCV design reduces this ambiguity and allows for a more direct interpretation of center-wise performance differences as indicators of domain shift.

From a clinical perspective, this type of evaluation is closer to the real deployment scenario: the model is trained on data from a set of known centers and then applied to a new center that contributes no labeled cases to the training process. The LoCoCV results therefore provide a realistic estimate of the performance drop that might occur when deploying the model at a new site.

We emphasize that the balanced LoCoCV design does not remove all sources of variability, but it reduces a major confounder by matching sample sizes across folds. In combination with reporting the mean ± standard deviation and the full Dice distributions, this provides a statistically transparent view of the uncertainty around center-wise performance under domain shift.

### 5.3. Interpretation of Center-Specific Patterns

Although the numeric values differ between centers, the general pattern is consistent: performance is the highest when the test distribution matches the training distribution, and performance decreases under domain shift. The detailed center-wise results also show that the benefit of three-phase input depends on the center.

- DUKE. In the random split, the three-channel model improves Dice from 0.446 to 0.528. Under LoCoCV, when DUKE is held out, Dice increases from 0.402 (1ch) to 0.433 (3ch), and HD95 decreases from 54.9 mm to 51.6 mm. This suggests that temporal enhancement information helps the model deal with DUKE-specific contrast and noise patterns.
- ISPY2. ISPY2 is the largest center and shows the highest Dice in the random split (0.687 and 0.729 for 1ch and 3ch). When ISPY2 is held out in LoCoCV, Dice drops to 0.469 (1ch) and 0.413 (3ch), and HD95 increases to about 41–46 mm. This indicates a strong domain shift between ISPY2 and the other centers and also shows that the three-channel model is not always more robust.
- NACT. At NACT, Dice is similar for 1ch and 3ch in both the random split and LoCoCV, while HD95 tends to increase slightly for 3ch. This center appears to be moderately affected by domain shift, with smaller changes than ISPY2.

Center-specific differences can arise from several factors:

- Scanner vendor, field strength, and coil design.
- Temporal resolution and contrast injection protocols.
- Reconstruction algorithms and noise characteristics.
- Differences in patient population, tumor histology, and the prevalence of multifocal disease.

Centers whose acquisition parameters are closer to the pooled training distribution will tend to show smaller performance drops in LoCoCV. Centers with more distinct acquisition characteristics are more likely to show larger drops. In real deployment, this means that model developers should not assume uniform performance across all sites in a network, even if the training dataset is multi-center.

### 5.4. Implications for Model Development and Deployment

The findings have several implications for how breast DCE-MRI segmentation models should be developed and validated:

- Need for explicit out-of-distribution testing. Evaluation based only on random splits is not sufficient when the intended use includes deployment to new centers. Center-wise or site-wise validation, such as LoCoCV, should become a standard part of model assessment.
- Multi-center training is necessary but not sufficient. Training on pooled data from several centers improves diversity, but it does not fully eliminate domain shift. Robustness to unseen centers remains a challenge even with multi-center training.
- Importance of reporting center-wise metrics. Aggregated metrics can hide poor performance on specific centers. Reporting the results per center, as in Table 2 and Table 3, helps identify where the model is likely to be safe and where additional adaptation or local validation is needed.
- Motivation for domain adaptation and harmonization. The performance gap between the random split baseline and the LoCoCV folds highlights the potential value of domain adaptation strategies, intensity harmonization, or scanner-aware models that explicitly account for acquisition differences.
- Choice of input representation. The comparison between the one-channel and three-channel models shows that temporal information is clearly beneficial in the optimistic random split setting but offers limited or center-dependent gains under LoCoCV. Future work should explore architectures that can exploit multi-phase information while remaining robust to cross-center variation.

### 5.5. Comparison with Recent Breast MRI Segmentation Studies

Several recent works have reported automatic breast MRI segmentation results on single-center or mixed-center data, often using U-Net variants or related architectures. For example, Refs. [10,11] describe 3D and 2D U-Net-based tumor and lesion segmentation on dynamic contrast-enhanced MRI from a single institution, reporting high Dice scores on internal train–validation–test splits. Other studies, such as [12,14], extend this line of work to weakly supervised tumor segmentation or whole breast segmentation on data from one or a few centers. Methods like those of [15,16] begin to explore robustness to protocol or scanner changes but still evaluate mainly on internal or two-domain settings. A summary of these studies is provided in Table 1. Our random split baseline on the multi-center MAMA-MIA dataset is broadly consistent with these prior reports. When training and testing on mixed-center data, the three-channel model reaches center-wise Dice values of 0.528 at DUKE, 0.729 at ISPY2, and 0.596 at NACT (Table 3). These values fall in the same general range as those in previous single-center DCE-MRI segmentation papers, despite the increased heterogeneity in MAMA-MIA. This suggests that, under an in-distribution evaluation protocol, a standard 3D U-Net can achieve performance that is competitive with more customized architectures reported in the literature. The main difference from most existing studies appears when we change the evaluation protocol from mixed-center random splits to balanced leave-one-center-out cross-validation. Under LoCoCV, the three-channel Dice drops to 0.433 at DUKE, 0.413 at ISPY2, and 0.480 at NACT (Table 2), that is, decreases of about 0.10 to 0.32 absolute Dice compared with the corresponding random split values. A similar pattern holds for the one-channel model. These drops are accompanied by increases in HD95, which indicate larger boundary errors on unseen centers. To our knowledge, most previous breast DCE-MRI segmentation studies do not report such balanced leave-one-center-out results, so the magnitude of this out-of-distribution penalty has remained largely hidden. Qualitative examples in Figure 8 further show that the failure modes under LoCoCV differ from the mixed-center setting. On the random split, errors are often limited to small boundary deviations, while on held-out centers, the model can underestimate tumor extent or miss satellite foci, especially at DUKE. These visual patterns align with the quantitative center-wise Dice and HD95 differences and illustrate how domain shift across centers affects practical segmentation quality. Overall, our results suggest that the segmentation accuracy reported in prior single-center or mixed-center studies may overestimate real-world performance when models are deployed across institutions. By combining a standard 3D U-Net baseline with a balanced leave-one-center-out design on MAMA-MIA, this work provides a transparent reference point that future methods, including more advanced architectures and domain adaptation techniques, can use when targeting robust multi-center breast DCE-MRI tumor segmentation.

### 5.6. Limitations and Future Work

This study has several limitations that suggest directions for future work.

- Single baseline architecture. We used a single 3D U-Net architecture with a fixed loss function and training setup. Although this is a strong and widely used baseline, other architectures may show different sensitivities to domain shift. Future work could compare 3D U-Net with transformer-based models, hybrid CNN–transformer designs, attention modules, and multi-scale or multi-task networks that jointly model tumor segmentation and related tasks such as breast or fibroglandular tissue segmentation.
- Fixed input representation. We evaluated two input configurations: a single-phase (1ch) and a three-phase (3ch) DCE representation. The results show that 3ch clearly helps in the random split setting but offers only center-dependent gains in LoCoCV. More flexible input strategies could be explored, such as learned temporal encodings, the parametric modeling of enhancement curves, or the selection of the most informative phases per center. It would also be useful to test whether combining DCE with other sequences (for example, T2-weighted MRI) changes the strength of domain shift.
- Limited center granularity. The analysis focused on three major center groups (ISPY2, DUKE, NACT) that have sufficient labeled pre-treatment DCE-MRI cases. In practice, each group can contain multiple scanners, protocols, and upgrades over time. Extending LoCoCV to finer scanner-level or protocol-level groupings, and adding more centers as they become available, could provide a more detailed map of domain shift across the full dataset.
- No explicit domain adaptation or harmonization. We deliberately did not apply explicit domain adaptation or intensity harmonization in order to first define a clear baseline. Future work could build on this baseline by adding domain adaptation modules, center-aware normalization, or intensity harmonization methods and then measuring how much they reduce the out-of-distribution performance penalty. Examples include adversarial domain adaptation, test time adaptation, style transfer-based harmonization, or simple scanner-specific calibration layers.
- Metric choices and clinical endpoints. The current evaluation used voxel-wise metrics such as Dice and HD95. These are important but do not capture the full clinical impact of segmentation errors. Future studies should include lesion-wise detection metrics, volume-based error analysis, and downstream endpoints such as response prediction, change in estimated tumor burden, or impact on treatment planning. Linking segmentation quality to clinical tasks would help translate the domain shift findings into concrete deployment guidelines.
- Sample size and annotation variability. Although ISPY2 and DUKE provide many cases, NACT has a smaller test set, which leads to wider confidence intervals for its metrics. In addition, all experiments used a single reference mask per case. Future work should quantify inter-observer variability, include multiple annotators when possible, and report confidence intervals for center-wise metrics, especially at smaller sites.

Despite these limitations, the present work provides a clear and reproducible baseline that quantifies how domain shift affects breast DCE-MRI tumor segmentation and demonstrates the value of balanced center-wise evaluation. The figures and metrics reported here can serve as a reference point for future studies on multi-center training, domain adaptation, and robust deployment of breast MRI segmentation models.

## 6. Conclusions

This paper investigated how domain shift across centers affects automated breast tumor segmentation in dynamic contrast-enhanced MRI using the multi-center MAMA-MIA dataset. We trained and evaluated a 3D U-Net under two complementary settings: a mixed-center random split that represents an optimistic in-distribution scenario and a balanced leave-one-center-out cross-validation (LoCoCV) setup that mimics deployment at a new hospital.

In the random split experiment, where cases from all three centers (ISPY2, DUKE, NACT) were pooled before splitting, both the one-channel and three-channel models converged stably. The three-channel model achieved a higher mean Dice and lower or similar HD95 at the main centers, and its Dice histogram was shifted toward higher values with fewer near-zero failures. Qualitative examples confirmed that temporal information from multiple DCE phases helps delineate enhancing tumors and reduces gross mis-segmentations when the training and test distributions match.

The LoCoCV experiments provided a more realistic test of generalization to unseen centers. When each center was held out in turn, the mean Dice decreased and HD95 increased compared with the random split baseline, even though the model was trained on a substantial number of cases from the other centers. Aggregating across folds, the one-channel and three-channel models achieved a very similar mean Dice (about 0.45) and comparable HD95, which shows that the main performance drop is driven by domain shift rather than by the choice of input representation. The center-wise results revealed that three-phase input can help at some sites (for example, DUKE) but may be neutral or even detrimental at others (for example, ISPY2), highlighting that multi-phase information is not automatically robust to cross-center variation.

To explain the different behaviors of the one-channel and three-channel models, we note that the three-phase DCE input gives the network more information about contrast dynamics, which explains the clear gain in the three-channel model in the mixed-center random split. However, the exact timing of the “early” and “late” post-contrast phases and the shape of the enhancement curves differ across centers. In retrospect, this behavior is consistent with how the dynamic series are acquired at the different centers. Under random splits, the model can exploit center-specific temporal patterns that are present in both training and test data. Under LoCoCV, the held-out center has different temporal sampling and enhancement dynamics, so these shortcuts no longer hold, and the three-channel model becomes more sensitive to protocol differences than the simpler one-channel model. In other words, the extra temporal information improves in-distribution performance but also increases vulnerability to inter-center variation in DCE-MRI timing.

Taken together, these findings support three main conclusions.

- Random split performance is optimistic. A model that performs well on mixed-center random splits can still show a substantial loss of accuracy when applied to a completely unseen center. Relying only on random splits may therefore overestimate real-world performance.
- Balanced LoCoCV is essential for deployment-oriented evaluation. By enforcing equal training, validation, and test sample sizes per center, the LoCoCV design separates domain shift effects from sample size effects and provides a more honest estimate of how performance will change when a model is deployed at a new site.
- Input representation and robustness are center-dependent. Three-phase DCE input improves performance in the optimistic random split setting and can help at some centers in LoCoCV, but it is not uniformly beneficial across all sites. This suggests that future methods should combine richer temporal modeling with explicit domain adaptation or harmonization to achieve robust cross-center performance.

From a clinical perspective, the results highlight that site-specific characteristics such as scanner vendor, protocol, and patient population can have a measurable impact on segmentation accuracy. Before deploying a breast DCE-MRI segmentation model in practice, it is therefore important to perform explicit cross-center validation, report center-wise metrics, and consider domain adaptation or local calibration when needed.

Future work should extend the present baseline by exploring alternative architectures, richer temporal encodings, explicit domain adaptation techniques, and clinically oriented evaluation endpoints. The experiments reported here provide a starting point and a quantitative reference for such studies, and they underscore that reliable multi-center validation is a necessary step toward the safe and reliable deployment of deep learning-based breast MRI segmentation models.

## Figures and Tables

**Table 1:** A summary of representative related work on breast MRI segmentation and domain shift and the remaining gap addressed in this study.

Work	Imaging Task	Dataset and Centers	Evaluation Design	Gap Versus This Study
[10]	Breast DCE-MRI tumor and lesion segmentation	Single-center DCE-MRI (one institution and scanner)	Random patient-wise split within the same center	Reports strong internal performance but does not test on unseen centers or analyze domain shift.
[11]	Lesion segmentation on early post-contrast images	Single-center DCE-MRI subtraction images (one institution)	2D U-Net with internal train/validation/test split from the same site	Focuses on internal accuracy with no multi-center evaluation or center-wise robustness analysis.
[12]	3D tumor segmentation with weak annotations	Breast DCE-MRI from one or a few centers (limited variability)	Internal evaluation with weak labels and standard random splits	Shows feasibility with weak labels but does not study performance on unseen centers or balanced center-wise cross-validation.
[14]	Whole breast segmentation for volumetric analysis	Breast MRI from one or a few centers (limited protocol variability)	Random splits mixing all available data	Targets whole breast segmentation (not primary tumor masks) and does not isolate domain shift across centers.
[15]	Fibroglandular tissue and background parenchymal enhancement segmentation in DCE-MRI	Data from one main scanner with some external scans	Attention U-Net with internal and limited external testing	Shows some robustness to scanner changes but focuses on parenchyma (not primary tumor segmentation) and uses limited center-wise analysis.
[16]	Cross-domain breast MRI segmentation with domain adaptation	Two domains (typically different populations or protocols)	Source–target setup with unsupervised domain adaptation and standard splits per domain	Uses advanced domain adaptation but in a narrow two-domain setting, without a large multi-center breast DCE-MRI dataset and balanced leave-one-center-out design.
[17]	Use of synthetic DCE-MRI for more robust lesion segmentation	Single- or few-center DCE-MRI plus simulated data	Internal evaluation with augmentation and random splits	Uses generative models to improve robustness but does not quantify center-wise domain shift on a multi-center benchmark.
[8]	Breast DCE-MRI tumor and non-mass enhancement segmentation; creation of multi-center benchmark	Multi-center DCE-MRI with several institutional groups and expert labels	Baseline models and challenge-style evaluation (often random or mixed-center splits)	Introduces MAMA-MIA, but early work mainly reports overall performance and does not perform a balanced leave-one-center-out analysis of primary tumor segmentation across major centers.
[18,19,20]	General medical imaging or neuroimaging segmentation and classification	Multi-site datasets with different scanners or protocols	Leave-one-site-out, leave-one-subject-out, and related strategies with some work on balanced folds	Highlight the importance of site-wise evaluation but do not apply a balanced leave-one-center-out design to breast DCE-MRI primary tumor segmentation on MAMA-MIA.

**Table 2:** Center-wise performance for the balanced LoCoCV experiment. Values are reported as the mean ± standard deviation over test lesions in the held-out center. The row “ALL” aggregates all three folds (DUKE, ISPY2, NACT).

Center	Dice (1ch)	Dice (3ch)	HD95 (1ch) [mm]	HD95 (3ch) [mm]	*n* (Test)
ALL	0.451±0.326	0.442±0.326	41.26±38.64	43.13±36.36	192
DUKE	0.402±0.356	0.433±0.354	54.89±48.34	51.62±44.99	64
ISPY2	0.469±0.318	0.413±0.322	41.23±34.98	45.71±33.26	64
NACT	0.481±0.295	0.480±0.295	28.95±26.06	32.71±26.71	64

**Table 3:** Center-wise performance for the random split experiment. Values are reported as the mean ± standard deviation over test lesions.

Center	Dice (1ch)	Dice (3ch)	HD95 (1ch) [mm]	HD95 (3ch) [mm]	*n* (Test)
DUKE	0.446±0.380	0.528±0.344	21.34±32.57	32.52±42.40	40
ISPY2	0.687±0.276	0.729±0.237	17.64±23.69	16.39±24.28	154
NACT	0.595±0.318	0.596±0.282	23.95±20.34	18.36±19.38	6

**Table 4:** Center-wise mean Jaccard (IoU), recall, and precision for random split experiment. Values are means over test lesions per center.

	1-Channel			3-Channel		
Center	Jaccard	Recall	Precision	Jaccard	Recall	Precision
DUKE	0.360	0.700	0.470	0.410	0.725	0.497
ISPY2	0.615	0.815	0.720	0.644	0.825	0.752
NACT	0.495	0.735	0.615	0.504	0.744	0.629

**Table 5:** Overall random split performance on mixed-center test set (200 lesions). Values are mean ± standard deviation, with median in parentheses, computed over test lesions.

Model	Dice	Jaccard (IoU)	Recall	Precision
1-channel	0.662±0.260 (0.780)	0.552±0.258 (0.640)	0.792±0.215 (0.882)	0.657±0.292 (0.775)
3-channel	0.695±0.253 (0.798)	0.581±0.255 (0.664)	0.805±0.207 (0.894)	0.681±0.287 (0.793)

## Data Availability

The breast DCE-MRI data used in this study are available as part of the public MAMA-MIA dataset from the challenge organizers. Processed metadata files and the configuration/training scripts used for this work are available from the corresponding author on reasonable request (and will be released in a public repository upon publication).

## References

[1] Keil V.C., Gielen G.H., Pintea B., Baumgarten P., Datsi A., Hittatiya K., Simon M., Hattingen E. (2021). DCE-MRI in glioma, infiltration zone and healthy brain to assess angiogenesis: A biopsy study. Clin. Neuroradiol..

[2] Zhao K., Dai P., Xiao P., Pan Y., Liao L., Liu J., Yang X., Li Z., Ma Y., Liu J. (2024). Automated segmentation and source prediction of bone tumors using ConvNeXtv2 Fusion based Mask R-CNN to identify lung cancer metastasis. J. Bone Oncol..

[3] Wang H., He Y., Wan L., Li C., Li Z., Li Z., Xu H., Tu C. (2025). Deep learning models in classifying primary bone tumors and bone infections based on radiographs. NPJ Precis. Oncol..

[4] Zhang C., Achuthan A., Himel G.M.S. (2024). State-of-the-art and challenges in pancreatic CT segmentation: A systematic review of U-Net and its variants. IEEE Access.

[5] Hall M.G., Cashmore M., Cho H.M., Ittermann B., Keenan K.E., Kolbitsch C., Lee C., Li C., Ntata A., Obee K. (2025). Metrology for MRI: The field you’ve never heard of. Magn. Reson. Mater. Physics Biol. Med..

[6] Ochi M., Komura D., Onoyama T., Shinbo K., Endo H., Odaka H., Kakiuchi M., Katoh H., Ushiku T., Ishikawa S. (2024). Registered multi-device/staining histology image dataset for domain-agnostic machine learning models. Sci. Data.

[7] Yousefirizi F., Klyuzhin I.S., O J.H., Harsini S., Tie X., Shiri I., Shin M., Lee C., Cho S.Y., Bradshaw T.J. (2024). TMTV-Net: Fully automated total metabolic tumor volume segmentation in lymphoma PET/CT images—A multi-center generalizability analysis. Eur. J. Nucl. Med. Mol. Imaging.

[8] Garrucho L., Reidel C., Kushibar K., Joshi S., Osuala R., Tsirikoglou A., Bobowicz M., del Riego J., Catanese A., Gwoździewicz K. (2025). A large-scale multicenter breast cancer DCE-MRI benchmark dataset with expert segmentations. Sci. Data.

[9] Ertas G., Doran S.J., Leach M.O. (2017). A computerized volumetric segmentation method applicable to multi-centre MRI data to support computer-aided breast tissue analysis, density assessment and lesion localization. Med. Biol. Eng. Comput..

[10] Khaled R., Vidal J., Vilanova J.C., Martí R. (2022). A U-Net Ensemble for breast lesion segmentation in DCE-MRI. Comput. Biol. Med..

[11] Douglas L., Bhattacharjee R., Fuhrman J., Drukker K., Hu Q., Edwards A., Sheth D., Giger M.L. (2023). U-Net breast lesion segmentations for breast dynamic contrast-enhanced magnetic resonance imaging. J. Med. Imaging.

[12] Park G.E., Kim S.H., Nam Y., Kang J., Park M., Kang B.J. (2024). 3D Breast Cancer Segmentation in DCE-MRI Using Deep Learning with Weak Annotation. J. Magn. Reson. Imaging.

[13] Jiao H., Jiang X., Pang Z., Lin X., Huang Y., Li L. (2020). Deep convolutional neural networks-based automatic breast segmentation and mass detection in DCE-MRI. Comput. Math. Methods Med..

[14] Narimani S., Hoff S.R., Kurz K.D., Gjesdal K., Geisler J., Grøvik E. (2025). Comparative analysis of deep learning architectures for breast region segmentation with a novel breast boundary proposal. Sci. Rep..

[15] Nowakowska S., Borkowski K., Ruppert C.M., Landsmann A., Marcon M., Berger N., Boss A., Ciritsis A., Rossi C. (2023). Generalizable attention U-Net for segmentation of fibroglandular tissue and background parenchymal enhancement in breast DCE-MRI. Insights Imaging.

[16] Kuang S., Woodruff H.C., Granzier R., van Nijnatten T.J.A., Lobbes M.B.I., Smidt M.L., Lambin P., Mehrkanoon S. (2023). MSCDA: Multi-level Semantic-guided Contrast Improves Unsupervised Domain Adaptation for Breast MRI Segmentation in Small Datasets. arXiv.

[17] Osuala R., Joshi S., Tsirikoglou A., Garrucho L., Pinaya W.H.L., Lang D.M., Schnabel J.A., Diaz O., Lekadir K. (2025). Simulating dynamic tumor contrast enhancement in breast MRI using conditional generative adversarial networks. J. Med. Imaging.

[18] Bradshaw T.J., Huemann Z., Hu J., Rahmim A. (2023). A Guide to Cross-Validation for Artificial Intelligence in Medical Imaging. Radiol. Artif. Intell..

[19] Pauli M.P., Pohl C., Golz M. (2021). Balanced Leave-One-Subject-Out Cross-Validation for Microsleep Classification. Curr. Dir. Biomed. Eng..

[20] Müller P., Kramer F. (2021). MIScnn: A framework for medical image segmentation with convolutional neural networks and deep learning. BMC Med. Imaging.

[21] Li X., Li L., Li M., Yan Y., Feng T., Luo H., Zhao Y., Yin S. (2025). Vision-Language Models in Medical Image Analysis: From Simple Fusion to General Large Models. Inf. Fusion.

[22] Li X., Li L., Li M., Yin S. (2025). Knowledge Distillation and Teacher-Student Learning in Medical Imaging: Comprehensive Overview, Pivotal Role, and Future Directions. Med. Image Anal..

